# The future of FemTech ethics & privacy – a global perspective

**DOI:** 10.1186/s12910-023-00976-z

**Published:** 2023-10-27

**Authors:** Najd Alfawzan, Markus Christen

**Affiliations:** 1 Institute of Biomedical Ethics and History of Medicine, University of Zurich, Winterthurerstrasse 30, 8006 Zürich, Switzerland; 2 Markus Christen Institute of Biomedical Ethics and History of Medicine & Digital Society Initiative, University of Zurich, Winterthurerstrasse 30, 8006 Zürich, Switzerland

**Keywords:** FemTech, Privacy, Ethics, Women’s Health, Digital Health, Roe v. Wade

## Abstract

We discuss the concept of women’s empowerment in FemTech, considering cultural and legal differences, ethical concerns, and legal consequences. We claim that it is crucial to prioritize privacy, a fundamental right, especially in the case of changes in laws related to women’s health, such as Roe v. Wade in the US.

## Background

The term ‘FemTech’ denotes technologies that are designed for women’s health. The FemTech industry has seen growth in recent years and is considered a rapidly expanding global market with a large user base [1]. However, studies conducted on FemTech have identified a number of privacy and security concerns related to the collection and use of personal and health data including the potential for unauthorized access, sharing, or misuse of this data, and the need for strong data privacy protection practices [2, 3]. This is of relevance given that FemTech companies must comply with diverging regulations and laws that exist in different countries. Furthermore, women’s health (WH) issues can vary in sensitivity depending on local cultural, religious, or legal factors.

## FemTech and empowerment

International human rights guidelines recognize a women’s right to make decisions about their own bodies and reproductive health (RH) [4]. This includes the right to make decisions about her body, the right to access reliable information about RH, and the right to reproductive freedom (RF) [5, 6]. The latter includes the right to access abortion and other RH services and the right to be free from discrimination and coercion when making decisions about RH [2].

FemTech can help women take control of their bodies and make informed decisions about their RH by providing accessible information on RH and convenient ways to track and manage their health. This can include a wide range of products and services, such as apps, devices, and services related to RH, fertility, pregnancy, menopause, and other WH issues [5].

Thus, FemTech has the potential to empower women in exercising their reproductive rights, as the FemTech industry claims repeatedly [7] – exemplarily shown by the FemTech Analytics Report of 2022: “FemTech is a technology that is empowering women” [1]. FemTech services are sold with the claim to give women more control and understanding of their bodies [5]. This supposed empowerment thus enables women to make informed choices about their menstrual, sexual, and RH and overall well-being [7].

## The sensitive nature of FemTech data

However, on the flipside of this claim to empowerment are the risks that FemTech may pose to its users. FemTech companies not only collect personal health data in the legal sense but also sensitive and intimate data in a cultural sense [8]. Such data can reveal sensitive and personal information about an individual; e.g., about an individual’s sexual preferences, sexual interactions, number of orgasms, birth control measures, childbearing journey, including miscarriage or abortion, and the associated mood and mental health of those women [2, 8].

If this intimate private data got shared, hacked, or mishandled, the affected women may suffer serious problems [2]. Although FemTech frequently gathers health data, it lacks the safeguards typically provided for health data collected in clinical settings. Because cultural and societal stigmas and taboos have historically relegated women’s health, bodies, and sexuality [5], mishandling this data can have significant implications for an individual’s privacy, safety, reputation, and overall well-being. Depending on the legal situation in the respective country, this could lead to legal surveillance, civil detentions, forced interventions, or criminal prosecution. This is why this data is considered to be highly sensitive and deserves protection – and the alarming level of women’s vulnerability to privacy risks due to the mismanagement and misuse of such data in a sociocultural context is a major concern [3].

## Diverse global regulations

Most of the FemTech companies offer their services globally, assisted by the global rise in smartphone usage. This means that they need to comply with a wide range of laws and regulations in different countries [5]. For assessing the consequences of this sensitive nature of FemTech data, we need to look at the diversity of global regulation – not only regarding data protection, but also concerning laws that govern women’s health, including laws around abortion.

In terms of privacy and data protection, FemTech companies must comply with different legal regimes. Some countries have specific laws that apply to health data, including data collected by FemTech, such as The EU General Data Protection Regulation [5]. In other countries, health data may be covered by more general data protection laws not specific to FemTech, such as the Health Insurance Portability and Accountability Act in the US [9]. Yet in other countries, there is even a lack of regulations when it comes to health data privacy [10]. This lack of a uniform privacy framework for health data broadly and FemTech data specifically has led to a wide range of privacy practices within the FemTech industry. This is of particular relevance for FemTech technologies specifically designed for women, who have historically faced oppression by social and legal systems [5].

When looking at differences related to laws that directly affect RF and health, the situation becomes even more complex. Laws and cultural norms around RH vary widely around the world [5]. In some countries, pregnancy outside of marriage is a criminal offense. In other countries, abortion is not legal, and even miscarriages can risk jail time. Countries like Iran have a law that severely restricts access to abortion, contraception, voluntary sterilization services, and related information [11]. Under such conditions, FemTech data may capture information on illegal activities, thus endangering users of FemTech should this data be accessed or shared. An illustrative case is the US, where on June 24, 2022, the Supreme Court overturned Roe v. Wade, ending almost 50 years of federally legal abortion [12, 13]. The overturn of Roe v. Wade allows states to make their own laws around abortion, leading to criminalization in some and liberalization in others. Where abortion is illegal, FemTech and other online data can then become an instrument to criminalize users [14–16]. As an example, in April 2017, a woman in Mississippi was accused of second-degree murder after experiencing a miscarriage and seeking treatment at a hospital. Prosecutors used her online search history and the purchase of the drug misoprostol as evidence to suggest that she had intentionally terminated her pregnancy [17]. In another example, the US Federal Trade Commission complaint revealed that the popular fertility tracking software, Premom by Easy Healthcare, shared users’ sensitive health information with third-party advertisers, including Google and the marketing firm AppsFlyer, without their consent since 2018 [18].

## Ethical problems

Given the complex nature of global regulations, especially when it comes to WH issues and data regulations, FemTech often fails to recognize that womanhood is not a one-size-fits-all experience, which only exacerbates the problems caused by the maintenance and reinforcement of norms in this area [5]. We suspect that FemTech providers assume that the data their tools collect cannot be used against their users. But this is wrong. Rather, the FemTech industry is confronted with the following three ethical problems that result from this diverse legal landscape.

We call the first ethical problem “deep misinformation” of the users of FemTech. As outlined above, this technology is embedded in a narrative of empowerment and liberation of women by collecting data and providing tools that allow the users to “take back control” and to even exercise a degree of freedom that may be in sharp contrast to the actual socio-cultural context in which the users are living. Users are not made aware that by using FemTech tools they may create “evidence” that can be used against them even in a legal sense.

The providers of FemTech are explicitly building their whole strategy of gaining users on the narrative of liberation and empowerment – maybe even reflecting their own “company philosophy” as well as their socio-cultural background, given that most such services are based in countries with relatively liberal regimes with respect to sexual and RH [7]. But when the promotion of those services does not consider that their users may live in countries whose social and legal systems are in conflict with such empowerment, we have a case of “deep misinformation”. Therefore, from an ethical point-of-view, the first minimal requirement for FemTech companies is to take the cultural context of their users into account in their informed consent policy to avoid this “deep misinformation”, even if this results in a loss of customers.

The second ethical problem concerns the potential “moral blindness” for those issues of the app developers, designers, and managers of the companies that create FemTech. They may indeed act in good faith by creating and promoting those technologies, but their view on the technology and the data collected by the technology is one-sided, neglecting the risk that a legislative change, as the case of overturning Roe v. Wade in the US illustrates, can create or that users living in more repressive legal or socio-cultural environments may bear. The absence of safeguards, such as the possibility to permanently delete data that may become dangerous for their users, engage developers in an ethical dilemma by making them accomplices of a policy that they may oppose. Therefore, from an ethical point-of-view, the second minimal requirement for FemTech companies is to include technological solutions that enable their users to detect that their FemTech device is collecting “dangerous data” and act upon it by creating enhanced safeguards.

The third ethical problem is that FemTech companies may be placed in a position that legally requires them to perform some “surveillance functions” if they want to be present in certain markets (e.g., by either routinely checking user data for suspicious behavior or by providing access to law enforcement authorities). Companies should be aware of this risk and proactively consider potential consequences. This may lead to the conclusion not to enter certain markets (such as Google who pulled its search engine from China in 2010 because of strict government censorship online) or to be prepared that their service may be blocked for users of certain countries not complying with certain law enforcement requests. Such decisions should then be explicitly outlined in the information policy for users from affected countries.

## Conclusion

Our considerations demonstrate that the nature of personal and health data collected by FemTech is intimate and deeply private. Furthermore, the legal conditions and regulations in various parts of the world are diverse – which may for example criminalize certain acts such as abortion – and they may become subject to significant changes over time. This contrasts with the global nature of FemTech, which is accessible across borders. Given that studies demonstrate rather poor data privacy and security standards of FemTech by sharing and/or selling data with third parties, we consider the following measures indispensable:

1. FemTech providers should inform the users that local legislation concerning RH may be in tension with the empowerment intention of the apps. They should point to the risk that certain kinds of data could become evidence against the user in criminal proceedings in countries where abortion, certain sexual preferences, or other aspects of reproductive and/or sexual health are criminalized.
2. FemTech providers should generally increase their data privacy and security standards. They should offer options that FemTech users can permanently delete their data if certain circumstances (such as legislative changes) endanger their users. They also should uphold the principle of data minimization and only collect data that is necessary for providing the service.
3. Femtech providers should be very restrictive in sharing any data they collect. Specifically, they should consider the possibility that they may be forced by law enforcement authorities to release data and they should implement preparatory measures to handle such cases to maximize the protection of their users.

Implementing those measures may be supported by creating a shared standard in the industry or may need more restrictive regulation. Nevertheless, we consider those measures necessary to uphold the stated intention of empowering women through FemTech and to protect the safety and security of the women FemTech companies claim to care for, no matter the legal, social, or cultural settings they live in.

## Data Availability

Not applicable.

## List of abbreviations

RF: Reproductive freedom
RH: Reproductive health
WH: Women’s health
